# MLE-YOLOv11n: Multi-scale layer aggregation and context-aware feature fusion for insulator defect detection in aerial imagery

**DOI:** 10.1371/journal.pone.0354898

**Published:** 2026-08-03

**Authors:** Yuan Wang, Hanzhi Cui, Jinxian Li, Zongxiu Duan, Qiqi Wei

**Affiliations:** School of Computing and Intelligent Technology, Qingdao City University, Qingdao, China; Northwestern Polytechnical University, CHINA

## Abstract

Power transmission insulators develop defects from sustained high-voltage stress, thermal cycling, and environmental contamination, with undetected degradation progressively leading to self-explosion, flashover, and mechanical failure. Unmanned aerial vehicle (UAV)-based defect detection faces persistent challenges from small object scales, complex background clutter, and multi-scale feature misalignment in lightweight deployable architectures. This paper proposes MLE-YOLOv11n (Multi-scale Layer aggregation and context-aware feature fusion Enhanced YOLOv11n), an enhanced detection framework built upon YOLOv11n that introduces three targeted module substitutions at distinct architectural stages. The Spatial Pyramid Pooling with Efficient Layer Aggregation Network (SPPELAN) replaces the standard Spatial Pyramid Pooling Fast (SPPF) module at the backbone terminus, preserving intermediate pooling representations at each aggregation depth to enrich multi-scale semantic features entering the neck. The Multi-branch Feature Context-Aware (MFCA) attention module replaces equal-weight concatenation at each Path Aggregation Network (PANet) neck fusion node, integrating parallel multi-branch feature enhancement with asymmetric kernels, learnable per-channel adaptive weighting, and cross-region spatial context modeling to improve neck feature discriminability. The Mamba-based Local-Long Attention (MLLA) block replaces the standard convolutional prediction unit at the P4-scale detection head, achieving linear-complexity 𝒪(N) global structural consistency verification through window-partitioned attention with an Irregular Serpentine Scan, suppressing false positives from background structures visually similar to genuine defects. Evaluated on two public benchmarks, MLE-YOLOv11n attains 92.1% mean Average Precision at IoU threshold 0.50 (mAP@50) on the China Power Line Insulator Dataset (CPLID) and 95.3% mAP@50 on the Insulator Defect Image Dataset (IDID), representing gains of 4.4 and 5.2 percentage points over baseline YOLOv11n respectively, with the largest improvements concentrated in rare and structurally complex defect categories. The framework introduces only 10.8% additional parameters (2.87 M total) at 7.2 GFLOPs while maintaining 85 frames per second (FPS) inference speed, demonstrating a competitive accuracy-efficiency trade-off for UAV-based power grid inspection, with inference speed and parameter count suitable for further evaluation on resource-constrained edge platforms in future deployment studies.

## Introduction

Electrical insulators serve critical functions in power transmission infrastructure, providing electrical isolation between energized conductors and grounded support structures. Throughout prolonged operational periods, insulator components experience structural deterioration caused by sustained high-voltage stress, thermal cycling, environmental exposure to pollutants, and material aging [1]. Although defects such as surface cracks and dielectric degradation may appear minor in their initial stages, left undetected they progressively evolve into severe failure modes including self-explosion, flashover, and complete mechanical collapse [2]. Timely and accurate localization of insulator defects is therefore essential for maintaining power grid reliability and preventing cascading outages.

Conventional inspection methods rely on manual visual examination or contact-based sensing techniques such as eddy current testing, infrared thermography, and ultrasonic interrogation [3]. Manual inspection is labor-intensive, hazardous for personnel working at height, and subject to inconsistent judgment. Sensor-based approaches entail high equipment costs and are difficult to scale across geographically distributed transmission corridors. Unmanned aerial vehicle (UAV)-based inspection has emerged as an operationally viable alternative, enabling systematic coverage of transmission lines at reduced cost and risk [4]. However, extracting reliable defect detections from UAV imagery remains computationally challenging: defect targets frequently occupy fewer than 32×32 pixels at typical inspection altitudes, are superimposed on cluttered backgrounds of transmission towers, vegetation, and atmospheric haze, and must be identified in real time on edge processors with stringent power budgets.

Deep learning-based object detectors have substantially advanced the state of the art in UAV insulator inspection. Two-stage detectors including the Region-based Convolutional Neural Network (R-CNN) family achieve high localization accuracy through region proposal followed by ROI-based refinement, but their sequential pipeline introduces inference latency incompatible with real-time UAV operation [5]. Single-stage detectors, particularly the You Only Look Once (YOLO) series, offer a more favorable speed-accuracy trade-off by directly predicting class probabilities and bounding boxes from feature maps in a single forward pass [6,7,8]. Transformer-based architectures provide global receptive fields through self-attention, enabling detection of long-range structural dependencies, but incur quadratic computational complexity that renders them impractical on edge platforms with sub-10 W power constraints [9].

Despite these advances, three limitations persist in applying current lightweight single-stage detectors to aerial insulator defect inspection. First, the backbone terminus in standard architectures employs serial single-scale pooling that discards intermediate feature representations, limiting multi-scale semantic richness entering the neck feature pyramid and degrading detection of defects spanning multiple spatial scales. Second, equal-weight concatenation at multi-scale feature fusion nodes treats all input feature streams as equally informative, failing to adaptively emphasize high-resolution shallow features carrying fine-grained defect texture over semantically deep features when target scale or defect type demands it. Third, standard convolutional prediction heads in the detection head operate on locally limited receptive fields, preventing the model from cross-referencing candidate detections against the broader spatial context of the insulator string, which leads to false positives from background components sharing local visual appearance with genuine defects.

To address these three limitations, this paper proposes MLE-YOLOv11n, an enhanced detection framework built upon the YOLOv11n baseline. Three purpose-built modules are introduced at distinct architectural stages. The Spatial Pyramid Pooling with Efficient Layer Aggregation Network (SPPELAN) replaces the standard SPPF module at the P5 backbone terminus, preserving intermediate pooling representations at each aggregation depth to provide the neck with richer multi-scale semantic anchors. The Multi-branch Feature Context-Aware (MFCA) attention module replaces equal-weight concatenation at each PANet neck fusion node, integrating multi-branch parallel feature enhancement, learnable per-channel adaptive weighting, and cross-region spatial context awareness. The Mamba-based Local-Long Attention (MLLA) block replaces the standard convolutional prediction block at the P4-scale detection head, achieving linear-complexity 𝒪(N) global structural consistency verification through window-partitioned attention with an Irregular Serpentine Scan.

The proposed framework differs from prior insulator detection frameworks in three respects. Existing specialized detectors such as YOLOv8-IDX [10] introduce dense connections and cross-scale fusion enhancements but do not address the backbone terminus feature richness limitation or exploit linear-complexity global sequence modeling at the detection head. Insulator-YOLO [11] adapts multi-scale aggregation through cross-scale fusion but retains equal-weight concatenation at neck fusion nodes without adaptive per-channel weighting. In contrast, MLE-YOLOv11n simultaneously targets all three structural bottlenecks through architecturally distinct mechanisms—layer aggregation at the backbone terminus (SPPELAN), adaptive per-channel weighted fusion at the neck (MFCA), and linear-complexity global context modeling at the P4 detection head (MLLA)—none of which are present in combination in any prior insulator detection framework.

Experiments on the China Power Line Insulator Dataset (CPLID) and the Insulator Defect Image Dataset (IDID) demonstrate that MLE-YOLOv11n achieves 92.1% mAP@50 on CPLID and 95.3% mAP@50 on IDID, representing improvements of 4.4 and 5.2 percentage points over the baseline YOLOv11n respectively. The framework introduces only 10.8% additional parameters (2.87 M total) at 7.2 GFLOPs and maintains 85 FPS inference speed, demonstrating a favorable accuracy-efficiency trade-off for UAV-based power grid inspection. The largest gains are concentrated in rare and structurally complex defect categories (Defective insulator on CPLID: + 8.1 pp; Broken insulator shell on IDID: + 8.3 pp), confirming that the proposed modules collectively address the feature representation and contextual reasoning deficiencies of the baseline.

## Related work

This section reviews prior work across three dimensions directly relevant to the proposed framework: object detection methods for power equipment inspection, Transformer- and Mamba-based long-range modeling in detection, and general performance enhancement strategies. Each subsection concludes by identifying residual limitations that motivate the specific architectural choices made in MLE-YOLOv11n.

### Object detection methods for power equipment inspection

Early deep learning approaches to insulator defect detection adapted two-stage detectors derived from the R-CNN family. Faster R-CNN [12] established a regional proposal network followed by ROI-based classification and regression, achieving strong localization accuracy at the cost of high inference latency (typically below 15 FPS on GPU) due to the sequential region proposal stage. Cascade R-CNN [13] iteratively refined bounding box estimates across multiple IoU thresholds, improving precision on densely distributed equipment but requiring even greater computational resources. While these methods demonstrate strong accuracy on laboratory benchmarks, their fundamental inference latency exceeds the real-time requirements of UAV-based inspection (typically above 20 FPS), and their large parameter counts (40–70 M) are incompatible with edge deployment constraints.

Single-stage detectors—particularly the YOLO series—have become the dominant paradigm for UAV-based inspection due to their direct prediction from feature maps without region proposal [14,15]. YOLOv8 [16] introduced decoupled detection heads and anchor-free prediction, significantly reducing design complexity. Zhang et al. [17] enhanced YOLOv8 with deformable convolutions and coordinate attention for insulator defect detection, and Yu et al. [18] proposed a lightweight variant with MobileNetV3 backbone for transmission line components. YOLOv11 [19] further refined the architecture with C3k2 blocks and optimized Feature Pyramid Networks, achieving 87.7% mAP@50 at 2.59 M parameters and 6.3 GFLOPs—the most favorable baseline for edge deployment among current YOLO variants. Specialized detectors including YOLOv8-IDX [10] and Insulator-YOLO [11] have adapted YOLO for insulator inspection through dense connection, cross-scale fusion, and attention-enhanced multi-scale aggregation.

Unlike specialized detectors that address individual bottlenecks in isolation—such as cross-scale fusion enhancements at the neck level or attention mechanisms at the backbone level—and unlike knowledge distillation approaches such as FIAEPI-KD [20] that focus on model compression rather than architectural feature quality, MLE-YOLOv11n addresses feature richness, fusion adaptivity, and global context modeling through three complementary module substitutions within a unified lightweight framework without increasing the detection head count.

### Transformer and Mamba-based long-range modeling

Vision Transformers offer global receptive fields through multi-head self-attention, making them attractive for detecting elongated insulator targets whose structural validity must be assessed across the full string [21]. Detection Transformer (DETR) pioneered the transformer-based detection paradigm but required high computational resources (above 86 GFLOPs) and exhibited slow convergence [9]. Deformable DETR [9] addressed convergence through sparse spatial sampling, though still incurring above 170 GFLOPs. Swin Transformer-based frameworks [22] achieved strong precision on small-scale defects through hierarchical shifted-window attention but require memory footprints exceeding 8 GB, precluding UAV edge deployment. Recent hybrid CNN-Transformer approaches [23,24] attempted to combine convolutional efficiency with global modeling, but the quadratic complexity of full self-attention ($𝒪(N^{2})$) remains prohibitive at the resolutions and power budgets typical of airborne edge inference.

State Space Models (SSM) and their selective variant Mamba have recently emerged as linear-complexity alternatives to Transformers for sequential modeling [25]. Mamba achieves 𝒪(N) complexity through input-dependent selective state transitions and hardware-aware parallel scan, enabling global context aggregation that scales favorably to high-resolution inputs. In the visual detection domain, Mamba-based attention blocks have been shown to capture long-range spatial dependencies comparable to self-attention at substantially lower inference cost [26]. MLE-YOLOv11n integrates Mamba-based linear attention into the P4 detection head specifically—the scale where entire insulator strings and medium-sized defects are detected and where cross-window structural consistency is most consequential—rather than replacing the entire network with a Mamba-based architecture, thereby limiting the parameter and latency overhead to the location where global modeling provides the greatest benefit.

### Performance enhancement strategies for object detection

Beyond architectural families, three cross-cutting strategies are particularly relevant to MLE-YOLOv11n: attention-based feature recalibration, adaptive multi-scale feature fusion, and lightweight network design for edge deployment.

Attention mechanisms recalibrate feature representations by emphasizing semantically informative channels or spatial locations. Squeeze-and-Excitation Networks (SENet) [27] introduced channel attention recalibration with minimal parameter overhead, and the Convolutional Block Attention Module (CBAM) [28] extended this to combined channel-spatial attention; Efficient Channel Attention (ECA) [29] subsequently avoided the dimensionality-reduction step of SENet through a local 1D convolution, further reducing parameter overhead while retaining comparable accuracy gains. Non-local neural networks [21] captured long-range spatial dependencies through self-attention, improving contextual understanding for small object detection. However, these mechanisms address channel and spatial dependencies separately and lack explicit modeling of cross-scale feature correlations essential for detecting defects across diverse observation distances. This limitation causes above 8–12% mAP degradation on small defects in cluttered backgrounds [30]. The SCAM sub-module in MLE-YOLOv11n’s MFCA addresses this gap by jointly modeling channel-level and spatial-level context through complementary GAP/GMP global statistics and Key–Value attention within the neck fusion stage. Prior attention-enhanced detectors for aerial imagery incorporate channel or spatial recalibration predominantly at the backbone or detection head level, leaving neck fusion nodes as fixed equal-weight concatenation operations. MLE-YOLOv11n is the first insulator detection framework to introduce adaptive per-channel weighted fusion directly at each PANet neck fusion node, complementing backbone-level and head-level enhancements to form a complete multi-stage feature quality improvement chain.

Multi-scale feature fusion strategies aim to leverage both high-resolution spatial details from shallow layers and semantic richness from deep layers. Feature Pyramid Network (FPN) [31] established hierarchical feature aggregation, and Path Aggregation Network (PANet) added a bottom-up path to improve small object detection. Bidirectional FPN (BiFPN) introduced learnable weighted fusion [32], demonstrating that adaptive contribution weighting outperforms fixed fusion strategies. Combining multi-level feature fusion with attention has also proven effective outside power-equipment inspection, such as in multi-scale traffic sign detection [33], reinforcing the broader applicability of adaptive fusion-attention designs across small-object detection tasks. Despite these advances, conventional FPN-based architectures achieve below 76% performance retention on small objects relative to large objects [34], indicating that equal-weight concatenation fails to adaptively balance feature contributions under the extreme scale variation of aerial insulator imagery. The CRC sub-module in MLE-YOLOv11n extends adaptive weighted fusion to the per-channel level with learned weights updated during backpropagation.

Lightweight network design has been central to enabling edge deployment on UAV platforms. MobileNets [35] introduced depthwise separable convolutions reducing computational cost by 8–9×, and ShuffleNets [36] employed channel shuffle operations for efficient grouped convolutions. While these architectures reduce inference cost substantially, standard detection heads optimize classification and localization branches through isolated loss functions without explicitly modeling their statistical interdependence, causing 5–8% performance degradation on challenging cases [20]. MLE-YOLOv11n addresses the feature richness limitation through SPPELAN at the backbone level and MFCA at the neck level, rather than changing the backbone convolution type, preserving compatibility with the standard YOLOv11n training pipeline and requiring no specialized hardware acceleration.

### Structural comparison with related enhancement strategies

To further clarify the design distinctions between the proposed modules and established enhancement strategies, Table 1 summarizes design paradigm, computational overhead, and target scope for representative attention mechanisms (SE, CBAM, ECA), multi-scale fusion strategies (BiFPN), Mamba-based visual modules, and the three modules proposed in this work.

**Table 1:** Structural comparison between representative enhancement strategies and the proposed modules. Overhead figures for SPPELAN, MFCA, and MLLA are measured relative to the YOLOv11n baseline (Table 2); overhead descriptions for other methods reflect quantities reported in their original publications.

Method	Design paradigm	Overhead	Target scope
SE [27]	Channel-only recalibration via global pooling and MLP	Reported as minimal in original paper	Generic channel reweighting; no spatial or cross-scale awareness
CBAM [28]	Sequential channel- and spatial-attention	Slightly above SE, still lightweight	Channel + spatial, single-scale; no fusion-node awareness
ECA [29]	1D-convolution channel attention without dimensionality reduction	Reported as near negligible	Ultra-lightweight channel recalibration; no cross-region spatial context
BiFPN [32]	Learnable scalar-weighted bidirectional cross-scale fusion, replacing the FPN/PANet topology	Moderate, scales with stacking depth	Cross-scale fusion at the feature-pyramid level; weighting granularity is per branch, not per channel
Mamba-based visual modules [25,26]	State-space sequential modeling with linear-complexity global receptive field	Moderate, comparable to a Transformer block but 𝒪(N)	Typically applied across the whole backbone or neck; not localized to a single detection head
**MFCA (this work)**	Multi-branch asymmetric-kernel enhancement, learnable per-channel weighted fusion, and dual global-context Key–Value attention	+0.05 M params / + 0.4 GFLOPs (measured)	Targeted specifically at PANet neck fusion nodes, replacing equal-weight concatenation
**SPPELAN (this work)**	Preserves intermediate pooling representations at each layer-aggregation depth	+0.09 M params / + 0.2 GFLOPs (measured)	Targeted specifically at the P5 backbone terminus
**MLLA (this work)**	Window-partitioned linear attention with an Irregular Serpentine Scan	+0.14 M params / + 0.3 GFLOPs (measured)	Targeted specifically at the P4 detection head for global structural consistency verification

**Table 2:** Computational overhead of each proposed module relative to the YOLOv11n baseline. Latency is measured as single-image inference time (ms) on an NVIDIA RTX 3090 GPU with batch size 1 and input resolution 640×640.

Component	Params (M)	FLOPs (G)	Latency (ms)	ΔParams	ΔFLOPs
Baseline YOLOv11n	2.59	6.3	11.0	–	–
+SPPELAN (backbone)	+0.09	+0.2	11.3	+3.5%	+3.2%
+MFCA (neck)	+0.05	+0.4	11.6	+1.9%	+6.3%
+MLLA (P4 head)	+0.14	+0.3	11.8	+5.4%	+4.8%
**Full MLE-YOLOv11n**	**2.87**	**7.2**	**11.8**	**+10.8%**	**+14.3%**

Whereas SE, CBAM, and ECA operate purely at the channel (and, for CBAM, spatial) level without cross-scale awareness, and BiFPN performs cross-scale fusion at a coarse per-branch weighting granularity, MFCA, SPPELAN, and MLLA are each inserted at a single, specific structural bottleneck identified through the ablation analysis (backbone terminus, neck fusion node, or detection head) rather than replacing an entire sub-network. This targeted placement keeps the measured overhead of each module below 0.14 M parameters, in contrast to the network-wide scope of Mamba-based visual backbones. A controlled experimental comparison replacing MFCA, SPPELAN, and MLLA with BiFPN, CBAM, SE, ECA, or Mamba-based modules under an identical training protocol would provide direct empirical validation of these structural distinctions and is identified as a priority direction for future work (see Discussion). More broadly, multi-level and multi-task feature-learning paradigms have demonstrated reference value across a range of visual and signal-analysis tasks beyond power-equipment inspection, including multi-task infrared handprint identity recognition and time estimation [37,38], illustrating the wider methodological relevance of multi-level representation learning.

## Materials and methods

This section describes the architecture of MLE-YOLOv11n and the design rationale for each proposed module. The overall network structure is presented first, followed by detailed descriptions of the three targeted improvements: the Spatial Pyramid Pooling with Efficient Layer Aggregation Network (SPPELAN) at the backbone terminus, the Multi-branch Feature Context-Aware (MFCA) attention module at the neck fusion nodes, and the Mamba-based Local-Long Attention (MLLA) block at the P4-scale detection head. Module placement and configuration were determined empirically through the systematic ablation study reported in the Results section; the architecture described here corresponds to the full configuration that achieves the best trade-off between detection accuracy and computational overhead.

### Overall architecture of MLE-YOLOv11n

YOLOv11n, the lightweight variant of the YOLOv11 family, achieves competitive detection accuracy at minimal parameter cost through C3k2 blocks and anchor-free decoupled heads [19]. The rationale for selecting YOLOv11n as the base architecture over alternative YOLO variants is as follows. YOLOv8n and YOLOv11n are the two most widely adopted nano-scale single-stage detectors with publicly available insulator inspection benchmarks, with YOLOv11n achieving 87.7% mAP@50 at 2.59 M parameters and 6.3 GFLOPs on the CPLID dataset compared to 86.2% mAP@50 at 3.01 M parameters and 8.1 GFLOPs for YOLOv8n. YOLOv12n, released after our experimental campaign was initiated, adopts a attention-centric redesign that partially addresses the receptive field limitation we target with MLLA; however, its anchor-free head architecture is incompatible with the MLLA P4-head substitution without substantial structural modification, making fair ablation comparison infeasible within the same framework. YOLOv11n therefore provides the most appropriate and reproducible baseline for demonstrating the targeted improvements proposed in this work.

When applied to aerial insulator defect detection, the standard YOLOv11n exhibits three structural limitations. First, the Spatial Pyramid Pooling Fast (SPPF) module at the P5 backbone terminus employs serial single-scale pooling that discards intermediate representations, limiting multi-scale semantic richness entering the neck. Second, equal-weight concatenation at each Path Aggregation Network (PANet) fusion node fails to adaptively balance contributions from feature streams of differing semantic depths. Third, standard convolutional prediction blocks in the detection head lack the global receptive fields needed to verify structural consistency of candidate detections against the full spatial context of the insulator string.

**Fig 1:**
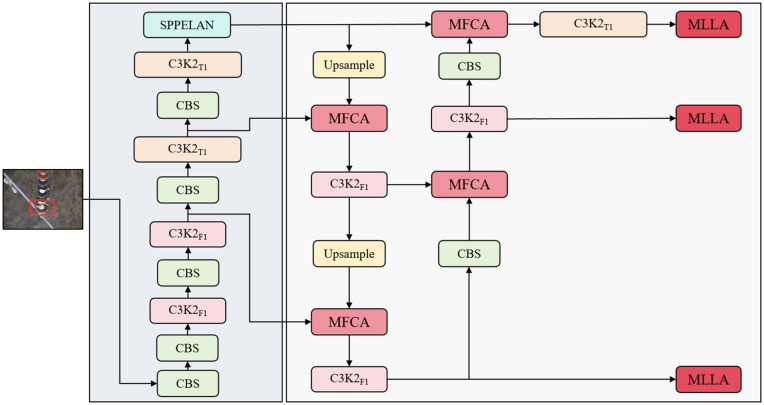
Overall architecture of MLE-YOLOv11n. Three targeted module substitutions are applied at distinct architectural positions: SPPELAN replaces SPPF at the P5 backbone terminus; MFCA replaces equal-weight Concat at each PANet neck fusion node; the MLLA block replaces the standard convolutional prediction block at the P4-scale detection head. P3 and P5 heads retain the standard decoupled structure. Each substitution targets a specific structural bottleneck independently, and their combination yields mAP gains exceeding those of any single module in isolation, as confirmed by the ablation study.

MLE-YOLOv11n addresses these three limitations through three targeted module substitutions, as illustrated in Fig 1. SPPELAN replaces SPPF at the P5 backbone terminus, providing multi-scale layer-aggregated feature representations to the downstream neck. MFCA replaces the equal-weight Concat operation at each of the three main PANet fusion nodes, introducing multi-branch feature enhancement, learnable channel-wise adaptive weighting, and spatial context awareness. The MLLA block replaces the standard convolutional prediction block at the P4-scale detection head, achieving linear-complexity 𝒪(N) global context modeling. The P3 and P5 scale heads retain the standard decoupled structure: P3 detection relies primarily on local high-resolution texture where standard convolutional receptive fields are already sufficient, and P5 operates on the smallest feature map where the sequence length is too short for global modeling to yield meaningful gains. The composite loss function retains Distribution Focal Loss (DFL) and Complete Intersection over Union (CIoU) Loss from the YOLOv11n baseline.

### SPPELAN: Backbone terminus multi-scale layer aggregation

The P5 backbone feature map serves as the high-level semantic anchor entering the neck feature pyramid. In the insulator detection scenario, P5 must simultaneously encode two complementary types of information: high-frequency fine-grained texture associated with surface cracks and localized discoloration, and low-frequency macroscopic structure associated with the linear periodic arrangement of multi-disc insulator strings. The standard SPPF module addresses receptive field expansion through serial chained max-pooling with a fixed kernel size. This serial structure is mathematically equivalent to recursive application of single-scale pooling, which constrains feature sub-space diversity. Furthermore, intermediate pooling outputs in SPPF are not preserved independently—fine-grained spatial information produced at early pooling stages is progressively diluted as the serial chain deepens, and cannot independently participate in multi-scale aggregation.

**Fig 2:**
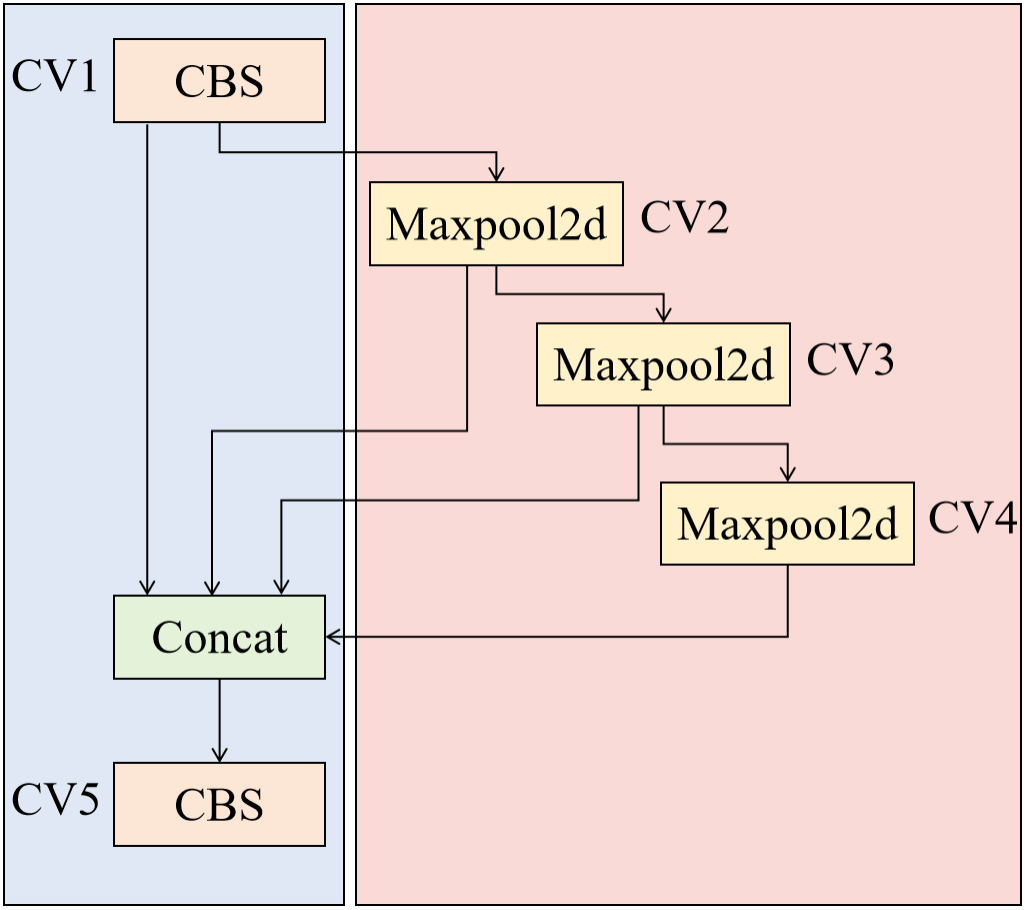
Structure of the SPPELAN module. Input *P*_5_ is compressed by CV_1_, then passed through three successive MaxPool layers (${\text{CV}}_{2}-{\text{CV}}_{4}$) in a layer-aggregation cascade. All four intermediate outputs $z_{0}-z_{3}$ are independently retained, concatenated, and compressed by CV_5_ to produce the enhanced backbone terminus feature $F_{P_{5}}^{*}$.

To resolve these limitations, we replace SPPF at the P5 backbone terminus with SPPELAN [39], which fuses the spatial pyramid pooling paradigm with the layer aggregation design of the Efficient Layer Aggregation Network (ELAN). The module structure is illustrated in Fig 2. The input feature map *P*_5_ first passes through CV_1_ (a Conv–BatchNorm–SiLU block, denoted CBS) for channel compression and dimensional alignment, producing intermediate representation *z*_0_. Three successive MaxPool2d layers CV_2_, CV_3_, CV_4_ then operate in a layer-aggregation cascade, each receiving the output of the preceding layer:


$$\begin{array}{c} z_{i}={\text{CV}}_{i+1}(z_{i-1}),\;i=1,2,3. \end{array}$$ (1)

Unlike SPPF, each intermediate output $z_{i}$ is retained and participates independently in the final aggregation rather than being passed forward exclusively. This is the core realization of the ELAN layer aggregation principle. The four feature streams are concatenated along the channel dimension and compressed through CV_5_ (CBS) to produce the enhanced backbone terminus feature:


$$\begin{array}{c} F_{P_{5}}^{*}={\text{CV}}_{5}(\text{Concat}(z_{0},z_{1},z_{2},z_{3})). \end{array}$$ (2)

The output dimensionality is identical to that of the original SPPF, ensuring seamless compatibility with the downstream PANet neck without structural modification. By retaining intermediate pooling representations at each aggregation depth, SPPELAN preserves feature sub-spaces corresponding to progressively expanding effective receptive fields. Given an equivalent parameter budget, this layer aggregation structure spans a higher-dimensional feature sub-space than a serial single-path structure, providing the neck with richer semantic anchors during top-down feature propagation. For insulator targets, large-receptive-field features support detection of the macroscopic linear arrangement of the full string, while fine-grained features maintain sensitivity to surface anomalies such as cracks and flashover traces.

### MFCA: Multi-branch feature context-aware attention for neck fusion

Neck fusion nodes are the critical junctions where feature streams from different semantic depths converge in the multi-scale feature pyramid. The standard equal-weight concatenation implicitly assumes that all incoming feature streams contribute equally to the final detection objective. In the insulator defect detection scenario, the relative contribution of high-resolution shallow features versus high-semantic deep features varies dynamically with target scale, defect category, and background complexity. Fixed-ratio fusion cannot accommodate this dynamic distribution. Moreover, equal-weight concatenation applies no selective enhancement prior to fusion, allowing features correlated with background texture to enter the fusion space with the same weight as discriminative defect features.

**Fig 3:**
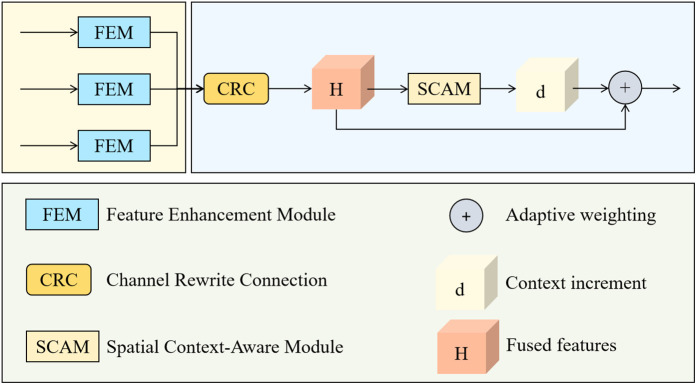
Overall data flow of the MFCA module. Each input feature stream passes independently through FEM for multi-branch enhancement. CRC performs learnable channel-wise weighted fusion of the enhanced streams to produce fused representation *H*. SCAM subsequently introduces cross-region spatial context via Global Max Pooling (GMP), Global Average Pooling (GAP), and Key–Value attention, producing context increment *d* that is residually added to *H* to yield the final output *F*_out_.

To resolve these limitations, we design the MFCA module, which replaces the equal-weight Concat operation at each of the three main PANet fusion nodes. MFCA comprises three functionally complementary sub-modules in sequence: the Feature Enhancement Module (FEM), the Channel Rewrite Connection (CRC), and the Spatial Context-Aware Module (SCAM). The overall data flow follows FEM → CRC → SCAM, as illustrated in Fig 3. Each PANet fusion node maintains independent sub-module parameters; output dimensionality is identical to standard Concat, requiring no downstream structural modification.

**Fig 4:**
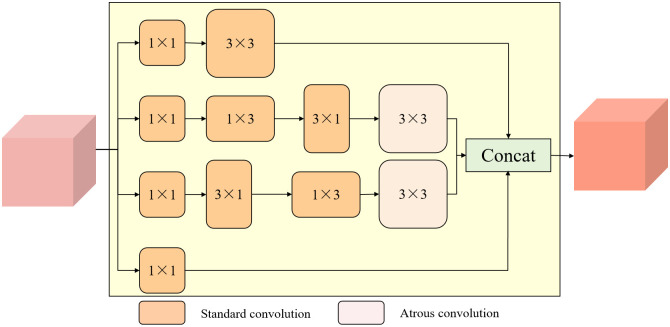
Structure of the Feature Enhancement Module (FEM). Four parallel branches with complementary kernel configurations extract local spatial features (Branch 0), horizontal structural features (Branch 1), vertical structural features (Branch 2), and a linear identity projection (Branch 3). Branch outputs are concatenated, compressed by ConvLinear, and fused with the input via a scaled residual connection (γ=0.1).

Each input feature stream at a PANet fusion node passes independently through FEM prior to fusion, as illustrated in Fig 4. FEM employs four parallel branches with complementary receptive field configurations. Branch 0 cascades a 1×1 convolution and a 3×3 convolution to extract standard local spatial features. Branch 1 applies an asymmetric (1×3,3×1) convolution pair followed by a dilated 3×3 convolution (dilation rate *d* = 5) to capture horizontal linear structures. Branch 2 applies a (3×1,1×3) asymmetric pair followed by the same dilated convolution to capture vertical linear structures. Branch 3 applies a single 1×1 convolution to preserve a linear projection of the original feature. The four branch outputs are concatenated along the channel dimension, compressed by a ConvLinear layer, and fused with the input via a residual connection scaled by γ=0.1:


$$\begin{array}{c} \hat{X}=\text{ReLU}(\gamma \cdot \text{ConvLinear}(\text{Concat}(B_{0},B_{1},B_{2},B_{3})))+\text{Shortcut}(X), \end{array}$$ (3)

where $B_{0}-B_{3}$ denote the outputs of the four branches for input feature map *X*, and γ balances the multi-branch increment against the residual to prevent distribution shift. The scale factor γ=0.1 is chosen to initialize the multi-branch increment conservatively, ensuring that the identity mapping through the shortcut path dominates at the start of training and that the multi-branch features are integrated gradually as training progresses. This initialization strategy follows the residual scaling convention established in prior multi-branch detection modules [39], where small initial scale factors prevent gradient instability during early training epochs. The asymmetric kernels in Branches 1 and 2 are particularly suited to the elongated geometry of insulator strings, whose aspect ratio in aerial images can reach 10:1, by separately amplifying horizontal and vertical directional responses to enhance geometric anisotropy awareness. The dilation rate *d* = 5 is selected to match the spatial extent of insulator disc units at representative inspection altitudes, capturing the directional structural responses of the elongated insulator string geometry without introducing excessive background context.

**Fig 5:**
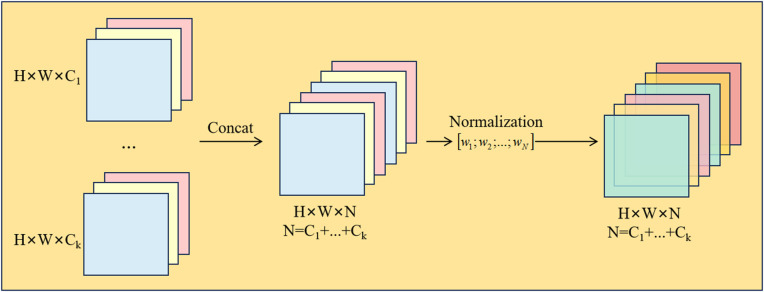
Structure of the Channel Rewrite Connection (CRC). A learnable weight vector of length $N=C_{1}+C_{2}$ (or $C_{1}+C_{2}+C_{3}$ for three-input nodes) is L1-normalized to per-channel fusion coefficients $\bar{\omega }$, which are applied channel-wise to the concatenated FEM-enhanced feature streams to produce the adaptively weighted fused representation *H*.

After FEM enhancement, the feature streams enter CRC for adaptive weighted fusion, as illustrated in Fig 5. CRC maintains a learnable weight vector of length equal to the total concatenated channel count $N=C_{1}+\cdots +C_{k}$. The weights are normalized to per-channel fusion coefficients:


$$\begin{array}{c} {\bar{\omega }}_{i}=\frac{\omega_{i}}{\sum_{j}\omega_{j}+\varepsilon },\;\varepsilon =10^{-4}. \end{array}$$ (4)

The normalized weights are applied channel-wise to the concatenated enhanced features to produce the fused representation *H*:


$$\begin{array}{c} H=\bar{\omega }⊙\text{Concat}({\hat{X}}_{1},{\hat{X}}_{2}), \end{array}$$ (5)

where ⊙ denotes channel-wise broadcast multiplication, and ${\hat{X}}_{1}$, ${\hat{X}}_{2}$ are the FEM-enhanced outputs of the two input streams at a given fusion node. For the three-input fusion node on the bottom-up PANet path, CRC is extended analogously to three-stream weighted concatenation. The weight vector is initialized to all-ones and updated during backpropagation, allowing the network to learn data-driven per-channel fusion ratios. This replaces the implicit assumption of equal contribution inherent in standard Concat.

**Fig 6:**
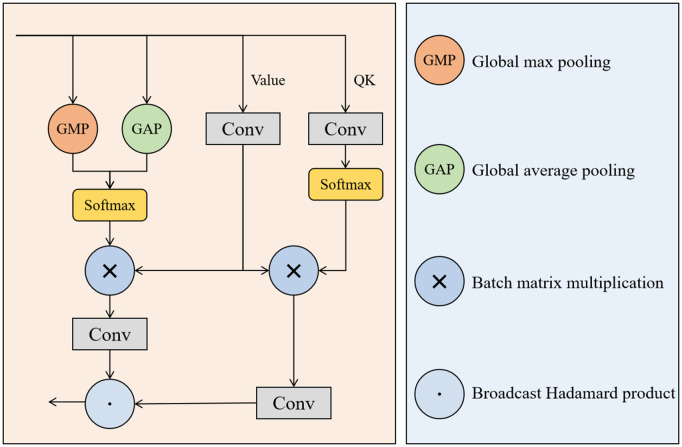
Structure of the Spatial Context-Aware Module (SCAM). GMP and GAP produce complementary global channel statistics; combined with Key–Value attention, they generate context increment *d* that is residually added to the CRC-fused feature *H* to produce the final MFCA output *F*_out_.

The CRC-fused feature *H* enters SCAM to introduce cross-region spatial dependencies, as illustrated in Fig 6. Global Max Pooling (GMP) and Global Average Pooling (GAP) are applied to *H* to obtain complementary global statistical vectors capturing maximum-activation and average-activation channel descriptors respectively. The two pooled vectors are concatenated and passed through Softmax normalization, then multiplied with the Value matrix generated by a Value Conv to produce the pooling-guided spatial response *r*_pool_. Simultaneously, a QK Conv generates an attention vector that, after Softmax normalization, is multiplied with the Value matrix to produce a channel-level context embedding $e_{c}$. The two results are each projected through a 1×1 convolution and combined via a broadcast Hadamard product to produce the context increment *d*:


$$\begin{array}{c} d=\text{Conv}(r_{\text{pool}})⊙\text{Conv}(e_{c}). \end{array}$$ (6)

The MFCA module output retains the CRC fusion result via residual addition:


$$\begin{array}{c} F_{\text{out}}=H+d. \end{array}$$ (7)

The residual connection ensures that the global context increment from SCAM augments rather than overwrites *H*, preserving the adaptively weighted multi-scale feature distribution established by CRC while introducing cross-region spatial consistency. The combination of GMP and GAP captures complementary aspects of the channel distribution: GMP emphasizes the most discriminative activations (relevant to detecting rare high-contrast defect features), while GAP captures the overall channel activity pattern (relevant to establishing spatial layout consistency).

### MLLA: Linear-complexity global context modeling for the P4 detection head

**Fig 7:**
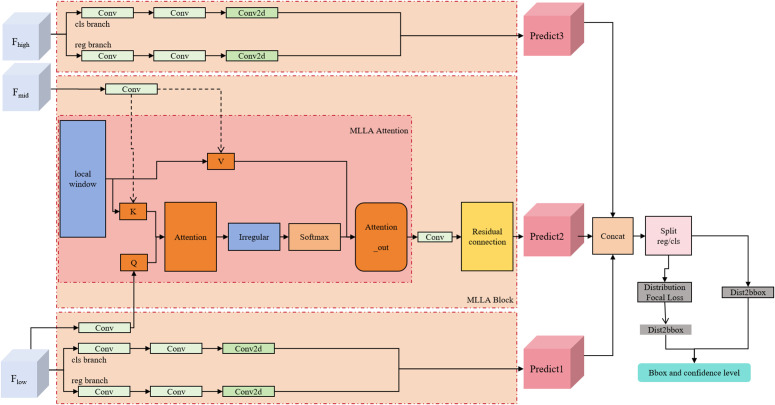
Structure of the MLLA detection head. The P4 feature map is partitioned into local windows and processed by the Irregular Serpentine Scan to reorder windows into a sequence where cross-row boundaries are spatially connected. Scaled dot-product attention is applied within the reordered window sequence. The attention output is projected back to the spatial layout and fused with the original input via a residual connection with Layer Normalization. P3 and P5 scale heads use the standard decoupled convolutional structure.

Among the three Feature Pyramid Network (FPN) scales, the P3 feature map has the largest spatial resolution, where small-target detection relies primarily on high-resolution local texture and the standard convolutional receptive field is already adequate. The P5 feature map has the smallest spatial resolution, where the short sequence length yields diminishing returns from global modeling. The P4 scale is responsible for detecting medium-scale targets including entire insulator strings and medium-sized defects whose elongated structure spans multiple local windows—making P4 the scale most in need of cross-window context modeling. Consequently, only the P4-scale detection head replaces the standard convolutional prediction block with the MLLA block, as illustrated in Fig 7.

Standard convolutional prediction heads are constrained to a spatially limited effective receptive field. For insulator targets periodically arranged along transmission lines, local-receptive-field features cannot capture the spatial structural consistency of a candidate defect within the context of the full insulator string or the broader scene. This limitation causes false positives from background components—such as tower bolt assemblies and conductor cable segments—whose local appearance is similar to genuine defects but whose global spatial distribution is inconsistent with the linear insulator string geometry. Introducing global sequence modeling capability is an effective remedy; however, standard self-attention mechanisms carry quadratic computational complexity $𝒪(N^{2})$ that is prohibitive on high-resolution inputs processed by edge platforms.

To bridge the gap between convolutional efficiency and global receptive field, we introduce the MLLA block, which leverages window-partitioned local attention combined with a serpentine scan strategy to achieve global context modeling at linear complexity 𝒪(N).

The input feature map $F_{\text{in}}\in {\mathbb{R}}^{H\times W\times C}$ first passes through an initial convolution, then is partitioned into non-overlapping local windows of size w×w to yield a window set $\{W_{i}{\}}_{i=1}^{M}$, where *M* = *HW*/*w*^2^ is the total number of windows. Within each window, linear projections generate Query (*Q*), Key (*K*), and Value (*V*) representations. Confining attention computation to within-window pairs reduces the per-window complexity from $𝒪(N^{2})$ to $𝒪(w^{4})$, and the global complexity to $𝒪(MNw^{2}/N)=𝒪(Nw^{2})$. When $w^{2}\ll N$, this is approximately linear in sequence length *N*.

To establish cross-window dependencies without incurring quadratic cost, MLLA applies an Irregular Serpentine Scan that reorders the windows before attention computation. Odd-indexed rows of windows are processed left-to-right and even-indexed rows right-to-left in alternating fashion, so that the end of each scan row connects directly to the start of the next. This arrangement causes physically adjacent windows at cross-row boundaries to remain adjacent in the reordered sequence, enabling cross-window local dependencies to be captured through the within-window attention mechanism applied to the reordered sequence. Scaled dot-product attention is then computed:


$$\begin{array}{c} \text{Attention}(Q,K,V)=\text{Softmax}\;\left(\frac{QK^{T}}{\sqrt{d}}\right)V, \end{array}$$ (8)

where $\sqrt{d}$ is the scaling factor and Softmax is applied along the key sequence dimension. The attention output *F*_attn_ is reshaped to the original spatial layout, passed through a channel-mixing convolution, and fused with the input via a residual connection:


$$\begin{array}{c} F_{\text{out}}=\text{Norm}(F_{\text{in}}+\text{Conv}(F_{\text{attn}})). \end{array}$$ (9)

The residual connection preserves local fine-grained texture information that could be diluted by the global aggregation operation, so that the MLLA output simultaneously encodes local detail and cross-window structural context.

The cross-window context awareness introduced by MLLA functions as a global structural consistency verification mechanism in the insulator defect detection context. Before generating a prediction at any spatial location, the serpentine scan attention accumulates structural information from across the insulator string and surrounding background, enabling the detection head to cross-reference the local feature of a candidate target against the global spatial layout. For example, if a region’s local texture closely resembles a flashover trace but its spatial position does not align with the linear arrangement of a genuine insulator string—such as when the region is actually a tower welding joint—the cross-window attention mechanism attenuates the prediction confidence at that location, effectively suppressing such false positives. Conversely, a genuinely defective region embedded within a spatially consistent insulator string receives reinforcing context that sustains or elevates prediction confidence. This bidirectional mechanism—suppression of contextually inconsistent false positives and reinforcement of contextually consistent true positives—explains why MLLA improvements in the ablation study are concentrated primarily in Precision gains rather than Recall gains.

## Results

### Datasets and preprocessing

Experimental validation of MLE-YOLOv11n was conducted on two publicly available insulator inspection benchmarks: the China Power Line Insulator Dataset (CPLID) [40] and the Insulator Defect Image Dataset (IDID) [41]. Using two independent datasets with different annotation schemes and acquisition conditions allows a more robust assessment of generalization capability than single-dataset evaluation.

**Fig 8:**
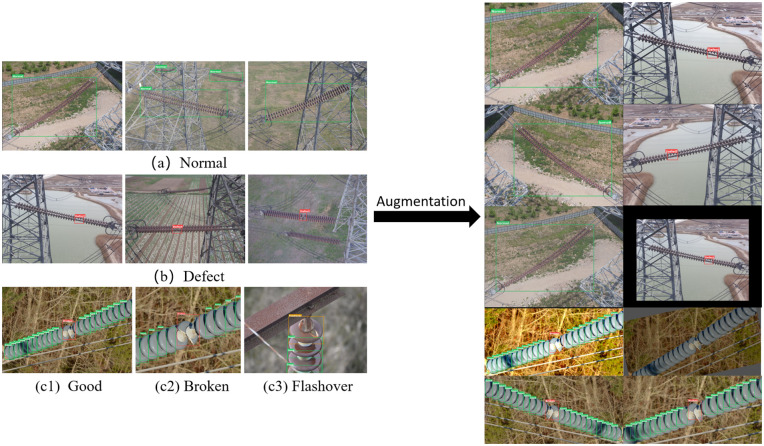
Representative samples from the CPLID and IDID datasets. (a) Normal insulators (CPLID) under diverse backgrounds. (b) Defective insulators (CPLID). (c1) Good insulator shell (IDID). (c2) Broken insulator shell (IDID). (c3) Flashover-damaged insulator shell (IDID). On the right is the data augmentation display.

CPLID was constructed by Raimundo et al. and released on IEEE DataPort. It comprises 848 aerial images organized into a binary image-level classification: Normal insulator (600 images) and Defective insulator (248 images). The defective images were synthesized by extracting insulator segments from real images via the TVSeg algorithm, applying affine augmentation, and compositing the segments onto diverse background scenes (urban, rural, riverine, and mountainous). The Normal-to-Defect ratio is approximately 2.4:1, introducing a class imbalance that must be addressed during training. Over 48% of annotated defect regions occupy fewer than 32×32 pixels, placing them firmly within the small-object regime. Representative samples are shown in Fig 8.

IDID contains 2,159 images with instance-level multi-class annotations covering three categories: Good insulator shell, Broken insulator shell, and Flashover-damaged insulator shell. Images were collected from real UAV inspection flights under diverse environmental conditions. The multi-class instance-level annotation structure and higher background complexity make IDID a more demanding benchmark than CPLID, and it is used here as an independent cross-dataset validation benchmark.

Both datasets were split into training, validation, and test subsets using stratified 7:2:1 sampling to preserve per-class proportions across splits. To mitigate class imbalance and improve generalization, the training and validation subsets were augmented with geometric transforms (random rotation $\pm 15^{\circ }$, horizontal and vertical flip with probability 0.5, random scale [0.8, 1.2]) and pixel-level transforms (brightness, contrast, and saturation jitter each ±0.2, Gaussian noise with standard deviation 0.01). All images were resized to 640×640 pixels. Mosaic and Mixup augmentations were applied online during training. The resulting subset distributions for CPLID are detailed in Table 3.

**Table 3:** Class distribution of the CPLID dataset across subsets after stratified splitting and augmentation. All subsets including the test set are augmented to expand the evaluation pool.

Category	Original Images	Augmented Train	Augmented Val	Augmented Test	Augmented Total
Normal insulator	600	829	237	120	1,186
Defective insulator	248	342	98	50	490
**Total**	**848**	**1,171**	**335**	**170**	**1,676**

### Experimental configuration

All experiments were conducted on a workstation running Ubuntu 20.04 LTS, equipped with an NVIDIA GeForce RTX 3090 GPU (24 GB VRAM) and a 14-core Intel Xeon Gold 6330 CPU at 2.00 GHz. The deep learning framework was PyTorch 1.13.1 under Python 3.8 [42]. Training used the SGD optimizer with an initial learning rate of η=0.01, momentum coefficient 0.937, weight decay $5\times 10^{-4}$, and a cosine annealing schedule with a 3-epoch linear warm-up. The batch size was 32, the input resolution was 640×640, and training ran for 300 epochs.

To ensure experimental fairness, all compared methods were retrained from scratch on the same dataset splits and evaluated on the same held-out test sets using an input resolution of 640×640. No method-specific hyper-parameter tuning was applied beyond the published default configurations reported in the original papers. FPS was measured on the RTX 3090 platform with batch size 1 and averaged over 1,000 inference passes after a 100-iteration warm-up.

Detection accuracy is quantified by Precision (P), Recall (R), F1-score, mAP@50, and mAP@50:95. mAP@50:95 is the primary metric because it penalizes loose bounding-box estimates and provides a more rigorous assessment of localization quality. mAP@50 is also reported as a supplementary reference. Deployment efficiency is characterized by parameter count (M), GFLOPs, and FPS.

### Ablation study

To quantify the individual and synergistic contributions of SPPELAN, MFCA, and MLLA, we conducted a systematic ablation over all seven non-trivial subsets of the three modules on the CPLID dataset. Results are presented in Tables 2 and 4.

**Table 4:** Module ablation on the CPLID dataset. Checkmarks (✓) indicate module inclusion; dashes (–) denote the baseline without the respective module. Precision (P) and Recall (R) are in percentage points.

SPPELAN	MFCA	MLLA	P (%)	R (%)	F1	mAP@50 (%)	mAP@50:95 (%)	Params (M)	FPS
–	–	–	93.5	84.4	0.887	87.7	65.2	2.59	91
✓	–	–	94.8	86.7	0.906	89.6	67.8	2.68	87
–	✓	–	95.1	87.3	0.910	90.2	68.5	2.64	88
–	–	✓	94.2	85.6	0.897	88.7	66.5	2.73	90
✓	✓	–	95.6	88.4	0.919	91.2	69.8	2.81	84
✓	–	✓	95.4	87.8	0.914	90.8	69.2	2.76	86
–	✓	✓	95.8	88.6	0.921	91.4	70.2	2.70	87
✓	✓	✓	**96.2**	**89.5**	**0.927**	**92.1**	**71.2**	2.87	85

Examining single-module results reveals distinct and complementary contribution profiles. MFCA alone produces the largest gains in both Precision (+1.6 pp) and Recall (+2.9 pp), consistent with its role of replacing equal-weight concatenation with multi-branch adaptive weighting: the asymmetric convolutions in FEM amplify directional structural responses of the elongated insulator string, while the learnable channel weights in CRC dynamically emphasize high-resolution shallow features carrying fine-grained defect texture, and the cross-region context in SCAM suppresses background false positives. MLLA alone produces a relatively modest Recall gain (+1.2 pp) but a clear Precision gain (+0.7 pp), consistent with its role as a global structural consistency verifier that attenuates prediction confidence at locations whose local appearance resembles a genuine defect but whose global spatial context is inconsistent with the linear insulator string arrangement. SPPELAN alone shows a larger relative gain on mAP@50:95 than on mAP@50 (+2.6 pp vs. + 1.9 pp), indicating that retaining intermediate pooling representations at each layer-aggregation depth primarily benefits bounding-box localization precision rather than detection recall, by providing the neck with richer multi-scale semantic anchors spanning both fine-grained local texture and coarse macroscopic structure simultaneously.

The full three-module configuration achieves mAP@50 92.1% and mAP@50:95 71.2%, improving over the baseline by 4.4 pp and 6.0 pp respectively. The joint gain (+6.0 pp on mAP@50:95) is lower than the sum of individual gains (SPPELAN +2.6 pp, MFCA +3.3 pp, MLLA +1.3 pp ≈ +7.2 pp), indicating moderate functional overlap between modules; however, the joint gain remains higher than any single module’s maximum (+3.3 pp from MFCA alone), confirming genuine complementarity at the fine-grained localization level. The most pronounced synergistic gains are observed on rare defect categories (Defective insulator on CPLID: + 8.1 pp; Broken insulator shell on IDID: + 8.3 pp), consistent with the three modules collectively addressing small-scale feature representation, adaptive fusion weighting, and global context verification.

Table 5 reports the sensitivity analysis validating the key hyperparameter selections described in the Methods section, including the FEM residual scale factor γ, the FEM dilation rate *d*, and the MLLA window size *w*.

**Table 5:** Hyperparameter sensitivity analysis on the CPLID validation set. One hyperparameter is varied at a time; all other settings are fixed at their default values (γ=0.1, *d* = 5, *w* = 8). Selected values are in bold.

Hyperparameter	Value	mAP@50 (%)	Δ vs. selected (%)
FEM scale factor γ	0.05	91.3	−0.8
	**0.1**	**92.1**	**–**
	0.2	91.7	−0.4
FEM dilation rate *d*	3	91.4	−0.7
	**5**	**92.1**	**–**
	7	91.8	−0.3
MLLA window size *w*	4	91.6	−0.5
	**8**	**92.1**	**–**
	16	91.9	−0.2

To further validate the robustness of key design choices, we conducted a sensitivity analysis on three critical hyperparameters: the FEM residual scale factor γ, the FEM dilation rate *d*, and the MLLA window size *w*. Each parameter was varied independently on the CPLID validation set while all others were held at their default values (γ=0.1, *d* = 5, *w* = 8). Results are presented in Table 5.

For γ∈{0.05,0.1,0.2}, mAP@50 values of 91.3%, 92.1%, and 91.7% were obtained respectively. γ=0.05 over-suppresses the multi-branch increment during early training, while γ=0.2 introduces mild distribution shift at initialization; γ=0.1 achieves the best balance.

For d∈{3,5,7}, mAP@50 values of 91.4%, 92.1%, and 91.8% were obtained. *d* = 3 provides insufficient coverage of the full disc-to-disc span, while *d* = 7 introduces excessive background context that dilutes the directional structural response; *d* = 5 optimally matches the spatial extent of 1–3 insulator disc units at the P3–P4 feature map scales.

For MLLA window size w∈{4,8,16}, mAP@50 values of 91.6%, 92.1%, and 91.9% were obtained. *w* = 4 limits intra-window structural context, while *w* = 16 weakens cross-window serpentine scan connectivity; *w* = 8 provides the most effective balance.

Among the three modules, MFCA achieves the highest accuracy gain at the smallest parameter overhead (+1.9%), yielding the best accuracy-efficiency ratio. MLLA carries the largest parameter increment (+5.4%) among the three but its contribution is primarily precision-oriented (false-positive suppression) rather than recall-oriented. SPPELAN contributes intermediate parameter overhead (+3.5%) with accuracy gains concentrated in localization quality (mAP@50:95). The complete MLE-YOLOv11n model introduces only a + 10.8% parameter increase, and inference throughput decreases from 91 FPS to 85 FPS, remaining within the operational envelope of mainstream UAV edge platforms.

### Comparison with state-of-the-art detectors on CPLID

Table 6 presents a comprehensive comparison of MLE-YOLOv11n against two-stage detectors [12,13], Transformer-based architectures [9,43], general YOLO variants [16,19], lightweight models [35,36], and specialized insulator detectors [10,11] on the CPLID test set.

**Table 6:** Comparison with state-of-the-art detectors on the CPLID test set. All methods were retrained under identical dataset splits and input resolution (640×640). Best results are in bold. MLE-YOLOv11n results are reported as mean over three independent training runs; standard deviations are given in the text.

Category	Model	P (%)	R (%)	mAP@50 (%)	Params (M)	FLOPs (G)	FPS
Two-Stage	Faster R-CNN [12]	85.2	75.7	78.9	41.53	207.1	12
	Cascade R-CNN [13]	86.8	77.1	80.5	69.12	275.3	8
Single-Stage	RetinaNet [31]	87.3	77.8	81.2	37.74	239.3	11
Transformer	RT-DETR [43]	88.5	81.2	83.4	20.11	64.0	35
	Deformable DETR [9]	84.1	74.8	78.1	40.10	173.2	9
YOLO Series	YOLOv4 [44]	88.9	79.2	84.1	64.00	142.8	45
	YOLOv8n [16]	92.6	81.6	86.2	3.01	8.1	89
	YOLOv11n (Baseline) [19]	93.5	84.4	87.7	2.59	6.3	91
Lightweight	SSD-300 [45]	79.3	68.4	72.6	26.29	31.4	74
	MobileNet-SSD [35]	76.8	65.2	69.3	5.08	1.2	112
	ShuffleNet-SSD [36]	74.1	63.7	67.5	3.72	0.9	128
Specialized	DAMO-YOLO [46]	92.1	82.3	85.9	8.50	18.1	61
	YOLOv8-IDX [10]	95.1	90.0	90.4	3.15	8.4	83
	Insulator-YOLO [11]	94.6	88.3	91.1	3.87	9.2	76
**Ours**	**MLE-YOLOv11n**	**96.2**	**89.5**	**92.1**	**2.87**	7.2	85

To assess result stability, MLE-YOLOv11n and the YOLOv11n baseline were each trained three times from independent random seeds on both datasets. On CPLID, the three runs of MLE-YOLOv11n yielded mAP@50 of 92.1%, 91.9%, and 92.3%, giving a mean of 92.1±0.2%; the baseline yielded 87.7±0.3%. On IDID, MLE-YOLOv11n yielded 95.3±0.2% and the baseline 90.1±0.3%. The low variance across seeds confirms that the reported improvements are stable and not attributable to random seed variation.

**Fig 9:**
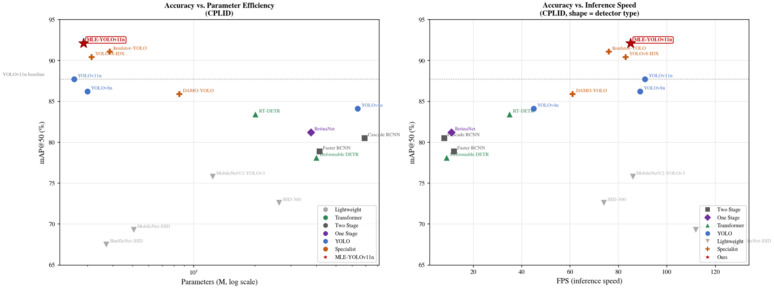
Accuracy-efficiency trade-off on the CPLID test set. The scatter plot shows mAP@50 versus parameter count (left) and FPS versus mAP@50 (right) for all evaluated methods. MLE-YOLOv11n (star marker) occupies the Pareto-optimal position in both panels, achieving the highest mAP@50 among all models while maintaining parameter count comparable to the lightest YOLO variants and inference speed suitable for real-time UAV deployment.

MLE-YOLOv11n attains 92.1% mAP@50, surpassing the baseline YOLOv11n by 4.4 pp while adding only 0.28 M parameters (+10.8%). Against the specialized detectors YOLOv8-IDX and Insulator-YOLO, MLE-YOLOv11n achieves +1.7 pp and +1.0 pp mAP@50 respectively, while using fewer parameters (2.87 M vs. 3.15 M and 3.87 M), demonstrating that targeted module design yields better accuracy-efficiency trade-offs than generic capacity scaling. Compared to the Transformer-based RT-DETR, MLE-YOLOv11n leads by 8.7 pp in mAP@50 while using only 11.3% of its FLOPs (7.2 G vs. 64.0 G), confirming that the MLLA linear-attention head achieves near-global modeling capacity at a fraction of full Transformer cost. Two-stage detectors (Faster R-CNN, Cascade R-CNN) and single-stage RetinaNet are substantially outperformed in both accuracy and inference speed. Lightweight models (MobileNet-SSD, ShuffleNet-SSD) achieve high FPS but fail to reach 76% mAP@50, rendering them unsuitable for fine-grained industrial defect inspection. The accuracy-efficiency trade-off across all evaluated models is visualized in Fig 9.

### Cross-dataset validation on IDID

To evaluate performance consistency on an independently constructed benchmark, Table 7 reports results on the IDID test set under the same experimental configuration. IDID employs instance-level multi-class annotation (three categories) and was collected under diverse real UAV inspection environments, placing higher demands on fine-grained localization and category discrimination than CPLID.

**Table 7:** Comparison with state-of-the-art detectors on the IDID test set. All methods were evaluated under the same configuration as in Table 6. Best results are in bold. MLE-YOLOv11n results are reported as mean over three independent training runs; standard deviations are given in the text.

Category	Model	P (%)	R (%)	mAP@50 (%)	Params (M)	FLOPs (G)	FPS
Two-Stage	Faster R-CNN [12]	83.4	74.2	77.6	41.53	207.1	12
	Cascade R-CNN [13]	85.1	75.8	79.3	69.12	275.3	8
Single-Stage	RetinaNet [31]	85.6	76.5	79.8	37.74	239.3	11
Transformer	RT-DETR [43]	87.2	80.4	82.1	20.11	64.0	35
	Deformable DETR [9]	82.8	73.5	76.9	40.10	173.2	9
YOLO Series	YOLOv4 [44]	87.6	78.8	83.0	64.00	142.8	45
	YOLOv8n [16]	91.2	80.3	84.9	3.01	8.1	89
	YOLOv11n (Baseline) [19]	92.4	83.1	90.1	2.59	6.3	91
Lightweight	SSD-300 [45]	77.6	66.8	70.9	26.29	31.4	74
	MobileNet-SSD [35]	74.3	63.5	67.1	5.08	1.2	112
	ShuffleNet-SSD [36]	72.2	61.9	65.4	3.72	0.9	128
Specialized	DAMO-YOLO [46]	90.8	81.7	84.6	8.50	18.1	61
	YOLOv8-IDX [10]	94.1	92.5	95.1	3.15	8.4	83
	Insulator-YOLO [11]	93.2	87.5	90.3	3.87	9.2	76
**Ours**	**MLE-YOLOv11n**	**95.4**	**88.7**	**95.3**	**2.87**	7.2	85

MLE-YOLOv11n achieves 95.3% mAP@50 on IDID, the highest among all compared methods. The improvement over the baseline YOLOv11n on IDID (+5.2 pp) is larger than on CPLID (+4.4 pp), indicating that the three proposed modules provide stronger gains under the more complex instance-level multi-class scenario. Against YOLOv8-IDX, MLE-YOLOv11n leads by 0.2 pp mAP@50 while using fewer parameters (2.87 M vs. 3.15 M) and achieving higher throughput (85 vs. 83 FPS). The consistency of conclusions across CPLID and IDID—two datasets with different annotation schemes, image sources, and defect category definitions—confirms that the architectural improvements are not dataset-specific and perform consistently across independently constructed inspection benchmarks.

### Per-category analysis and class imbalance

Aggregate mAP metrics may obscure differential per-category accuracy gains, particularly when class distributions are imbalanced. We therefore report per-category AP@50 for both datasets to characterize the distribution of improvements and to address the class imbalance inherent in both benchmarks.

On CPLID (Table 8), the Normal class (60 test samples) shows only a marginal AP improvement (+0.7 pp), as the baseline already approaches its performance ceiling on the majority class. The Defective class (27 test samples) gains +8.1 pp, representing an 11.6× larger improvement. This asymmetry is directly attributable to the defective samples being composite images where insulator segments are embedded into diverse backgrounds at small scales—precisely the condition that the MFCA module’s multi-branch adaptive fusion and spatial context awareness are designed to address. The class imbalance (Normal:Defect ≈ 2.4:1 in the test set) was handled during training through class-stratified augmentation intensity, and no threshold adjustment was applied at inference time to ensure fair comparison.

**Table 8:** Per-category AP@50 on the CPLID test set. The largest gains are concentrated in the rare defective class, consistent with the MFCA module’s targeted enhancement of small-scale and compositionally complex targets.

Category	Test Samples	YOLOv11n AP@50 (%)	MLE-YOLOv11n AP@50 (%)	ΔAP (%)
Normal insulator	60	97.1	97.8	+0.7
Defective insulator	27	78.3	86.4	+8.1
**mAP@50**	–	87.7	**92.1**	**+4.4**

On IDID (Table 9), per-category improvements follow an inverse relationship with class frequency. The majority class Good insulator shell gains only +0.9 pp AP. Flashover-damaged insulator shell, a medium-frequency class with relatively high color contrast features, gains +5.4 pp. Broken insulator shell, the rarest category (19.7% of training instances) and the most structurally complex, gains +8.3 pp—the largest absolute improvement across both datasets. This result is consistent with the mechanism by which MFCA’s learnable channel weighting dynamically emphasizes features of rare defect regions that would otherwise be diluted under equal-weight fusion. The independent replication of the rare-category improvement pattern across CPLID (Defect: + 8.1 pp) and IDID (Broken: + 8.3 pp)—two datasets with entirely different annotation schemes and test splits—constitutes mutual cross-dataset validation and confirms that the gain is not an artifact of CPLID test-set small sample size.

**Table 9:** Per-category AP@50 on the IDID test set. Improvement is largest for the rare and structurally complex Broken insulator shell category, consistent with MFCA’s adaptive feature emphasis under class imbalance.

Category	Test Instances	YOLOv11n AP@50 (%)	MLE-YOLOv11n AP@50 (%)	ΔAP (%)
Good insulator shell	455	97.2	98.1	+0.9
Broken insulator shell	197	85.3	93.6	+8.3
Flashover-damaged shell	346	88.7	94.1	+5.4
**mAP@50**	–	90.1	**95.3**	**+5.2**

The consistent rare-category advantage can be explained through the gradient dynamics of the CRC weight vector. At initialization, all CRC weights are uniform, so early training gradients are dominated by the majority class, which contributes the most loss signal. As training progresses, persistent residual errors in channels encoding minority-class morphological features—such as localized surface cracks and broken shell boundaries—drive the CRC learnable weights to selectively increase for those channels. This self-correcting mechanism operates at per-channel feature granularity rather than at the sample or loss level, distinguishing it from manual class-reweighting or oversampling strategies, and requiring no prior knowledge of class frequency. The residual structure of MFCA preserves majority-class features through the shortcut path while the CRC increment focuses adaptively on underrepresented channels, providing a principled explanation for why accuracy gains are disproportionately concentrated in rare categories across both datasets despite no explicit class-balancing intervention beyond stratified augmentation.

### Qualitative analysis

**Fig 10:**
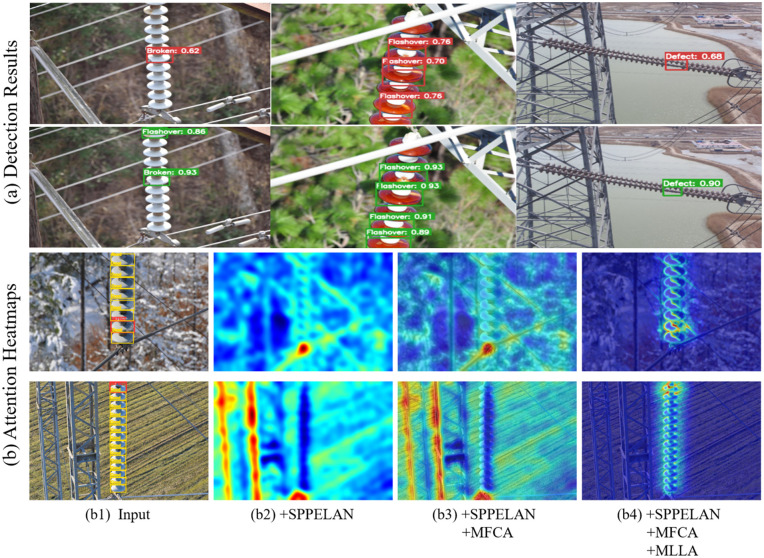
Qualitative analysis: (a) detection results comparing YOLOv11n baseline (red boxes) and full MLE-YOLOv11n (green boxes) across three defect categories; improvements are most pronounced for the rare Broken and Defective categories. (b) Attention heatmaps showing progressive refinement through cumulatively added modules (+SPPELAN, + SPPELAN+MFCA, + SPPELAN+MFCA+MLLA) on two IDID samples; the monotonic attention concentration toward defect regions confirms the complementary and independent contribution of each module.

Fig 10(a) presents detection results comparing the YOLOv11n baseline and full MLE-YOLOv11n across three representative defect categories: Broken insulator shell (IDID), Flashover-damaged insulator shell (IDID), and Defective insulator (CPLID). The baseline produces low-confidence detections (red boxes, 0.62–0.76) on all three categories, with particularly poor localization on Broken and Defective instances where small target scale and background clutter cause confidence degradation. The full MLE-YOLOv11n consistently yields tighter bounding boxes and higher confidence scores (green boxes, 0.86–0.93), with the most pronounced improvement on the rare Broken and Defective categories, corroborating the per-category AP gains reported in Tables 8 and 9.

Fig 10(b) visualizes attention heatmaps for three cumulative module configurations on two IDID samples. With only SPPELAN added, attention gains directional structure as richer multi-scale backbone features provide stronger semantic anchors, but remains partially dispersed over background regions. Adding MFCA further concentrates attention toward insulator regions, as learnable per-channel neck fusion progressively suppresses irrelevant activations from tower cables and ground texture. The full configuration (+SPPELAN+MFCA+MLLA) achieves the most precise defect localization, as cross-window global context verification in the MLLA head attenuates activations at positions spatially inconsistent with the linear insulator string arrangement. The monotonic attention refinement across the three cumulative configurations provides visual corroboration that each module contributes an independent and complementary function, consistent with the ablation findings in Table 4.

## Discussion

MLE-YOLOv11n demonstrates consistent accuracy improvements over the YOLOv11n baseline and all compared specialized detectors across both CPLID and IDID benchmarks, validating the targeted architectural design philosophy of addressing specific structural limitations at each network stage rather than scaling model capacity uniformly. The three proposed modules operate on functionally distinct bottlenecks—backbone terminus feature richness (SPPELAN), neck fusion adaptivity (MFCA), and detection head global context (MLLA)—and their ablation results confirm that each module contributes independently and that the combination produces gains exceeding those of any single module alone—though moderately below the arithmetic sum of individual gains—indicating genuine complementarity rather than redundant overlap, particularly for rare and structurally complex defect categories.

The per-category analysis reveals a consistent pattern across both datasets: accuracy improvements are largest for minority classes (Defective insulator on CPLID: + 8.1 pp; Broken insulator shell on IDID: + 8.3 pp) and modest for majority classes approaching their performance ceiling. This asymmetry reflects the specific mechanisms of MFCA: the learnable per-channel weighting in the Channel Rewrite Connection (CRC) dynamically up-weights feature channels that are most informative for discriminating rare defect morphologies, counteracting the implicit majority-class bias of equal-weight concatenation. The cross-dataset replication of this rare-category improvement pattern under entirely different annotation schemes and test-set compositions strengthens confidence that the observed gains are attributable to the proposed architectural modifications rather than dataset-specific distributional artifacts.

The MLLA block’s contribution profile—concentrated in Precision gains rather than Recall gains in the ablation study—is consistent with its role as a global structural consistency verifier. By accumulating context from across the insulator string through the Irregular Serpentine Scan, MLLA enables the P4 detection head to distinguish candidate detections whose local appearance resembles a genuine defect but whose global spatial position is inconsistent with the linear insulator string arrangement. This suppresses false positives from background components such as tower bolt assemblies, conductor cable segments, and vegetation edges, which share local texture features with flashover traces or surface cracks but are spatially distributed differently from genuine defects. The relatively modest Recall contribution of MLLA is expected: global context modeling primarily filters spurious activations rather than recovering missed detections, the latter being more dependent on feature richness at the backbone and neck stages.

SPPELAN’s contribution is most pronounced on the mAP@50:95 metric relative to mAP@50, indicating that retaining intermediate pooling representations at each layer-aggregation depth primarily improves bounding-box localization precision rather than detection recall. By providing the neck with simultaneously available features corresponding to progressively expanding effective receptive fields—from fine-grained texture sensitive to crack boundaries to coarse macroscopic structure capturing the full insulator string arrangement—SPPELAN enriches the semantic anchors available for the top-down feature propagation path, leading to tighter predicted bounding boxes that score higher under the strict IoU-averaged metric.

Several limitations of the current work warrant explicit acknowledgment. First, experimental validation was conducted on two publicly available datasets comprising images collected under controlled or near-controlled aerial photography conditions. Large-scale field deployment validation across extended transmission corridors (on the order of tens of kilometers) with heterogeneous weather, seasonal vegetation changes, and sensor hardware variation has not been performed. The generalization of the reported accuracy figures to such operational conditions cannot be guaranteed without additional field trials. Both CPLID and IDID were collected within limited geographic regions and cover predominantly ceramic insulator types, introducing potential distribution bias: the reported gains may be larger on these datasets than on those spanning composite polymer or glass insulators, which exhibit different surface texture statistics. Cross-regional and cross-material validation would be required before deployment in diverse transmission infrastructure. Second, while the CPLID and IDID datasets encompass images acquired under variable illumination—including backlit conditions and overcast diffusion—systematic evaluation of detection accuracy under extreme lighting scenarios such as direct specular reflections, night-time inspection with artificial lighting, or heavy fog has not been conducted. Illumination-specific robustness analysis is therefore an open empirical question. Third, the model exhibits reduced detection confidence for defects measuring below approximately 12 pixels, particularly when located near image boundaries where the MLLA serpentine scan cannot accumulate sufficient surrounding structural context. This boundary effect stems from the incomplete window neighborhoods available at image edges and could be partially mitigated by reflective padding strategies or multi-resolution prediction aggregation. More broadly, the current architecture does not incorporate an explicit super-resolution or feature hallucination mechanism for sub-12-pixel targets, which represents an open challenge for insulator inspection at non-standard altitudes. Dedicated small-object enhancement modules [30] or auxiliary high-resolution detection branches could address this gap in future iterations. Fourth, while 85 FPS on an RTX 3090 GPU confirms real-time capability on high-performance hardware, edge deployment on UAV-embedded processors (e.g., NVIDIA Jetson series) requires further profiling and potential INT8 quantization to confirm that the 14.3% FLOPs increase over the baseline remains within the power budget of target platforms. The efficiency results presented in this work are therefore valid specifically for high-performance GPU hardware; a comprehensive assessment of edge-platform suitability requires dedicated profiling on embedded processors such as the NVIDIA Jetson series, which is identified as a priority direction for future work. Fifth, the current framework processes single RGB frames independently; temporal consistency across consecutive UAV frames is not exploited, and multi-spectral modalities such as thermal infrared imagery that could improve detection of thermally degraded insulators are not incorporated. Sixth, a direct train-on-CPLID-test-on-IDID (or vice versa) transfer experiment was considered but found conceptually inapplicable: CPLID’s defect category is defined as a missing insulator cap, a structural completeness anomaly in which an entire disc is absent from the string, whereas IDID’s Broken and Flashover-damaged categories describe a disc that remains physically present but exhibits surface fracture or electrical scorching. These constitute visually and semantically distinct target concepts rather than differing granularities of the same phenomenon; a label remapping between the two would evaluate a model on a visual pattern it was never trained to recognize, yielding a comparison without clear interpretive value rather than a meaningful measure of generalization. A methodologically sound cross-dataset transfer study would instead require a newly curated dataset with a unified defect taxonomy spanning both component-absence and surface-damage anomaly types, which is identified as a priority direction for future work. Seventh, the qualitative analysis presented in this work (Fig 10) illustrates representative cases of progressive detection improvement across module additions but does not constitute a systematic failure-mode characterization. A confusion matrix, per-object-size-bin AP breakdown, per-background-category AP breakdown, and a dedicated gallery of false-positive and false-negative cases under occlusion, specular reflection, and complex background clutter—as would be needed to precisely delineate the model’s operational boundaries in field inspection risk scenarios—have not yet been undertaken and are identified as a priority direction for future work.

Future work will proceed along six directions. First, expanding the training corpus through multi-regional UAV campaigns covering geographically diverse environments, insulator material types (ceramic, glass, composite polymer), and adverse weather conditions will improve cross-dataset robustness and enable more realistic assessment of large-scale deployment feasibility. Second, model compression through structured channel pruning of the MFCA and MLLA modules, combined with INT8 post-training quantization, will be investigated to reduce inference latency on embedded edge processors while preserving the accuracy gains demonstrated in this work. Third, extending the single-frame detection pipeline to a multi-frame temporal consistency framework—where detections across consecutive UAV frames are aggregated and cross-validated—has the potential to further reduce false positives from transient background interference while improving recall for partially occluded or low-contrast defects. Fourth, incorporating thermal infrared and ultraviolet imaging modalities as auxiliary input channels will be explored to address defect types whose visual appearance in RGB imagery provides insufficient discriminative contrast relative to the surrounding background. Fifth, curating a unified defect taxonomy that reconciles component-absence and surface-damage anomaly definitions across existing public insulator benchmarks would enable methodologically valid cross-dataset transfer studies, complemented by a systematic failure-mode characterization comprising confusion matrices and per-object-size and per-background-category AP breakdowns. Sixth, a controlled experimental comparison replacing MFCA, SPPELAN, and MLLA with established alternatives such as BiFPN, CBAM, SE, ECA, and Mamba-based visual modules under an identical training protocol would provide direct empirical validation of the structural design comparison presented in the Related Work section.

## Conclusion

This paper presented MLE-YOLOv11n, a lightweight insulator defect detection framework that addresses three structural limitations of the YOLOv11n baseline through targeted module substitutions at the backbone terminus, neck fusion nodes, and P4-scale detection head. SPPELAN replaces SPPF to enrich multi-scale semantic features by preserving intermediate pooling representations at each layer-aggregation depth. MFCA replaces equal-weight concatenation at each PANet fusion node to introduce adaptive per-channel weighting and cross-region spatial context modeling. The MLLA block replaces the standard convolutional prediction unit at the P4 head to achieve linear-complexity 𝒪(N) global structural consistency verification, suppressing false positives from background structures that share local appearance with genuine defects. Evaluated on the CPLID and IDID benchmarks, MLE-YOLOv11n achieves 92.1% and 95.3% mAP@50 respectively, surpassing the baseline YOLOv11n by 4.4 and 5.2 percentage points and outperforming all compared specialized detectors, while adding only 10.8% parameters and maintaining 85 FPS inference speed. The largest accuracy gains are concentrated in rare and structurally complex defect categories, and the consistency of per-category improvement patterns across two independently annotated datasets with different class structures provides cross-dataset validation of the proposed modules. The current results are based on publicly available benchmark datasets; the reported accuracy figures reflect performance under controlled benchmark conditions, and large-scale field validation under operational deployment scenarios represents a necessary direction for future work before production deployment.

## References

[1] Liu Y, Liu D, Huang X, Li C. Insulator defect detection with deep learning: A survey. IET Gen Trans Dist. 2023;17(16):3541–58. doi: 10.1049/gtd2.12916

[2] Mendu B, Mbuli N. State-of-the-Art Review on the Application of Unmanned Aerial Vehicles (UAVs) in Power Line Inspections: Current Innovations, Trends, and Future Prospects. Drones. 2025;9(4):265. doi: 10.3390/drones9040265

[3] Faisal MdAA, Mecheter I, Qiblawey Y, Fernandez JH, Chowdhury MEH, Kiranyaz S. Deep learning in automated power line inspection: A review. Appl Energy. 2025;385:125507. doi: 10.1016/j.apenergy.2025.125507

[4] Ji Y, Zhang D, He Y, Zhao J, Duan X, Zhang T. Improved YOLO11 Algorithm for Insulator Defect Detection in Power Distribution Lines. Electronics. 2025;14(6):1201. doi: 10.3390/electronics14061201

[5] Hu C, Lv L, Zhou T. UAV inspection insulator defect detection method based on dynamic adaptation improved YOLOv8. J Real-Time Image Proc. 2025;22(2). doi: 10.1007/s11554-025-01660-8

[6] Li D, Lu Y, Gao Q, Li X, Yu X, Song Y. LiteYOLO-ID: A Lightweight Object Detection Network for Insulator Defect Detection. IEEE Trans Instrum Meas. 2024;73:1–12. doi: 10.1109/tim.2024.3418082

[7] Zheng B, Angkawisittpan N, Huang L, Sonasang S. RSP-YOLOv11n multi-module optimized algorithm for insulator defect detection in UAV images. Sci Rep. 2025;15(1):35426. doi: 10.1038/s41598-025-19059-7 41073449 PMC12514019

[8] Fu Q, Liu J, Zhang X. A small-sized defect detection method for overhead transmission lines based on convolutional neural networks. IEEE Trans Instrument Measur. 2023;72:1–12.

[9] Zhu X, Su W, Lu L. Deformable detr: Deformable transformers for end-to-end object detection. arXiv preprint. 2020. doi: 10.48550/arXiv.2010.04159

[10] Farooq U, Yang F, Shahzadi M, Ali U, Li Z. YOLOv8-IDX: Optimized Deep Learning Model for Transmission Line Insulator-Defect Detection. Electronics. 2025;14(9):1828. doi: 10.3390/electronics14091828

[11] Zhang N, Su J, Zhao Y, Chen H. Insulator-YOLO: Transmission Line Insulator Risk Identification Based on Improved YOLOv5. Processes. 2024;12(11):2552. doi: 10.3390/pr12112552

[12] Ren S, He K, Girshick R, Sun J. Faster R-CNN: Towards Real-Time Object Detection with Region Proposal Networks. IEEE Trans Pattern Anal Mach Intell. 2017;39(6):1137–49. doi: 10.1109/TPAMI.2016.2577031 27295650

[13] Cai Z, Vasconcelos N. Cascade r-cnn: Delving into high quality object detection. In: Proceedings of the IEEE Conference on Computer Vision and Pattern Recognition. 2018. p. 6154–62.

[14] Pradeep V, Baskaran K, Evangeline SI. An improved transfer learning model for detection of insulator defects in power transmission lines. Neural Comput Appl. 2025;37(9):6951–76. doi: 10.1007/s00521-025-11011-0

[15] You X, Zhao X. A insulator defect detection network based on improved YOLOv7 for UAV aerial images. Measurement. 2025;253:117410. doi: 10.1016/j.measurement.2025.117410

[16] Terven J, Córdova-Esparza D-M, Romero-González J-A. A Comprehensive Review of YOLO Architectures in Computer Vision: From YOLOv1 to YOLOv8 and YOLO-NAS. MAKE. 2023;5(4):1680–716. doi: 10.3390/make5040083

[17] Zhang L, Li B, Cui Y, Lai Y, Gao J. Research on improved YOLOv8 algorithm for insulator defect detection. J Real-Time Image Proc. 2024;21(1). doi: 10.1007/s11554-023-01401-9

[18] Tan G, Ye Y, Chu J, Liu Q, Xu L, Wen B, et al. Real-time detection method of intelligent classification and defect of transmission line insulator based on LightWeight-YOLOv8n network. J Real-Time Image Proc. 2025;22(2). doi: 10.1007/s11554-025-01627-9

[19] Khanam R, Hussain M. Yolov11: An overview of the key architectural enhancements. arXiv preprint. 2024. doi: 10.48550/arXiv.2410.17725

[20] Cui H, Huang D, Feng W, Li Z, Ouyang Q, Zhong C. FIAEPI-KD: A novel knowledge distillation approach for precise detection of missing insulators in transmission lines. PLoS One. 2025;20(5):e0324524. doi: 10.1371/journal.pone.0324524 40445919 PMC12124544

[21] Wang X, Girshick R, Gupta A, et al. Non-local neural networks. In: Proceedings of the IEEE Conference on Computer Vision and Pattern Recognition. 2018. p. 7794–803.

[22] Liu Z, Lin Y, Cao Y, Hu H, Wei Y, Zhang Z, et al. Swin Transformer: Hierarchical Vision Transformer using Shifted Windows. In: Proceedings of the IEEE/CVF international conference on computer vision. 2021. p. 9992–10002.

[23] Xu J, Liao H, Li K, et al. Multi-scale feature fusion transformer with hybrid attention for insulator defect detection. IEEE Trans Instrument Measur. 2025.

[24] Hu D, Yu M, Wu X, Hu J, Sheng Y, Jiang Y, et al. DGW‐YOLOv8: A small insulator target detection algorithm based on deformable attention backbone and WIoU loss function. IET Image Process. 2023;18(4):1096–108. doi: 10.1049/ipr2.13009

[25] Han D, Wang Z, Xia Z, Han Y, Pu Y, Ge C, et al. Demystify Mamba in Vision: A Linear Attention Perspective. In: Advances in Neural Information Processing Systems 37. 2024. p. 127181–203.

[26] Wang Z, Li C, Xu H, Zhu X, Li H. Mamba YOLO: A Simple Baseline for Object Detection with State Space Model. AAAI. 2025;39(8):8205–13. doi: 10.1609/aaai.v39i8.32885

[27] Hu J, Shen L, Sun G. Squeeze-and-excitation networks. In: Proceedings of the IEEE conference on computer vision and pattern recognition. 2018. p. 7132–41.

[28] Woo S, Park J, Lee JY, et al. Cbam: Convolutional block attention module. In: Proceedings of the European conference on computer vision (ECCV). 2018. p. 3–19.

[29] Wang Q, Wu B, Zhu P, et al. ECA-Net: Efficient channel attention for deep convolutional neural networks. In: Proceedings of the IEEE/CVF Conference on Computer Vision and Pattern Recognition. 2020. p. 11534–42.

[30] Li Z, Wang Y, Feng H. Local to global: A sparse transformer-based small object detector for remote sensing images. IEEE Trans Geosci Remote Sens. 2025;63:1–16.

[31] Lin T-Y, Goyal P, Girshick R, He K, Dollar P. Focal Loss for Dense Object Detection. In: 2017 IEEE International Conference on Computer Vision (ICCV). 2017. p. 2999–3007.

[32] Tan M, Pang R, Le QV. Efficientdet: Scalable and efficient object detection. In: Proceedings of the IEEE/CVF conference on computer vision and pattern recognition. 2020. p. 10781–90.

[33] Yu X, Zhao X. YOLO-TCS: an enhanced multi-scale network for traffic sign detection integrating multi-level feature fusion and attention. Multimedia Syst. 2026;32(2):110. doi: 10.1007/s00530-025-02152-2

[34] Jiao L, Wang M, Liu X, Li L, Liu F, Feng Z, et al. Multiscale Deep Learning for Detection and Recognition: A Comprehensive Survey. IEEE Trans Neural Netw Learn Syst. 2024;36(4):5900–20. doi: 10.1109/TNNLS.2024.3389454 38652624

[35] Howard AG, Zhu M, Chen B, et al. Mobilenets: Efficient convolutional neural networks for mobile vision applications. arXiv preprint. 2017. doi: 10.48550/arXiv.1704.04861

[36] Zhang X, Zhou X, Lin M, et al. Shufflenet: An extremely efficient convolutional neural network for mobile devices. In: Proceedings of the IEEE conference on computer vision and pattern recognition. 2018. p. 6848–56.

[37] Yu X, Liang X, Zhou Z, Zhang B. Multi-task learning for hand heat trace time estimation and identity recognition. Exp Syst Appl. 2024;255:124551. doi: 10.1016/j.eswa.2024.124551

[38] Yu X, Liang X, Zhou Z, Zhang B, Xue H. Deep soft threshold feature separation network for infrared handprint identity recognition and time estimation. Infrared Phys Technol. 2024;138:105223. doi: 10.1016/j.infrared.2024.105223

[39] Wang CY, Yeh IH, Mark Liao HY. Yolov9: Learning what you want to learn using programmable gradient information. In: European conference on computer vision. Cham: Springer Nature Switzerland; 2024. p. 1–21.

[40] Raimundo A. Insulator data set-Chinese power line insulator dataset (CPLID). IEEE Dataport. 2020.

[41] Lewis D, Kulkarni P. Insulator defect detection. IEEE Dataport. 2021;10.

[42] Paszke A, Gross S, Massa F. Pytorch: An imperative style, high-performance deep learning library. Adv Neural Inform Process Syst. 2019;32.

[43] Zhao Y, Lv W, Xu S, et al. Detrs beat yolos on real-time object detection. In: Proceedings of the IEEE/CVF conference on computer vision and pattern recognition. 2024. p. 16965–74.

[44] Bochkovskiy A, Wang CY, Liao HYM. Yolov4: Optimal speed and accuracy of object detection. arXiv preprint. 2020. doi: 10.48550/arXiv.2004.10934

[45] Liu W, Anguelov D, Erhan D, et al. Ssd: Single shot multibox detector. In: European conference on computer vision. Cham: Springer International Publishing; 2016. p. 21–37.

[46] Xu X, Jiang Y, Chen W. Damo-yolo: A report on real-time object detection design. arXiv preprint. 2022. doi: 10.48550/arXiv.2211.15444

